# The Landscape of Human Proteins Interacting with Viruses and Other Pathogens

**DOI:** 10.1371/journal.ppat.0040032

**Published:** 2008-02-15

**Authors:** Matthew D Dyer, T. M Murali, Bruno W Sobral

**Affiliations:** 1 Genetics, Bioinformatics, and Computational Biology Program, Virginia Polytechnic Institute and State University, Blacksburg, Virginia, United States of America; 2 Virginia Bioinformatics Institute, Virginia Polytechnic Institute and State University, Blacksburg, Virginia, United States of America; 3 Department of Computer Science, Virginia Polytechnic Institute and State University, Blacksburg, Virginia, United States of America; Pennsylvania State University, United States of America

## Abstract

Infectious diseases result in millions of deaths each year. Mechanisms of infection have been studied in detail for many pathogens. However, many questions are relatively unexplored. What are the properties of human proteins that interact with pathogens? Do pathogens interact with certain functional classes of human proteins? Which infection mechanisms and pathways are commonly triggered by multiple pathogens? In this paper, to our knowledge, we provide the first study of the landscape of human proteins interacting with pathogens. We integrate human–pathogen protein–protein interactions (PPIs) for 190 pathogen strains from seven public databases. Nearly all of the 10,477 human-pathogen PPIs are for viral systems (98.3%), with the majority belonging to the human–HIV system (77.9%). We find that both viral and bacterial pathogens tend to interact with hubs (proteins with many interacting partners) and bottlenecks (proteins that are central to many paths in the network) in the human PPI network. We construct separate sets of human proteins interacting with bacterial pathogens, viral pathogens, and those interacting with multiple bacteria and with multiple viruses. Gene Ontology functions enriched in these sets reveal a number of processes, such as cell cycle regulation, nuclear transport, and immune response that participate in interactions with different pathogens. Our results provide the first global view of strategies used by pathogens to subvert human cellular processes and infect human cells. Supplementary data accompanying this paper is available at http://staff.vbi.vt.edu/dyermd/publications/dyer2008a.html.

## Author Summary

Many pathogens, such as viruses and bacteria, cause disease in humans. Pathogen infections result in illness and death for millions of people each year. Pathogens communicate with human cells through physical interactions with various human proteins on the surface of the cell and within the interior of the cell. These interactions allow the pathogen to enter the host cell, manipulate important cellular processes, multiply, and invade other cells. In this paper, we compare interactions between human and pathogen proteins from 190 different pathogens to provide important insights into strategies used by pathogens to infect human cells. We show that both viral and bacterial proteins interact with human proteins that themselves interact with many human proteins or with human proteins that lie on many communication channels between other human proteins. Pathogens may have evolved to interact with these human proteins since they may control critical human cellular process. We also demonstrate that many viruses share common infection strategies, e.g., lengthening particular stages of the cell cycle, controlling programmed cell death, and interacting with the nuclear membrane to transfer viral genetic material into and out of the nucleus. Such studies may help us better understand the process of infection and identify better strategies to prevent or cure infection.

## Introduction

Infectious diseases result in millions of deaths each year. Millions of dollars are spent annually to better understand how pathogens infect their hosts and to identify potential targets for therapeutics. An important aspect of any host-pathogen system is the mechanism by which a pathogen is able to invade a host cell. Within these complex systems, protein-protein interactions (PPIs) between surface proteins form the foundation of communication between a host and a pathogen and play a vital role in initiating infection [1]. PPI-mediated mechanisms of infection have been studied in detail for many pathogens [2–7]. However, many questions are relatively unexplored. What are the properties of human proteins that interact with pathogens? Do pathogens interact with certain functional classes of human proteins? Which infection mechanisms and pathways are commonly triggered by multiple pathogens? A significant hurdle to such global cross-pathogen comparisons has been the shortage of large-scale datasets of interactions between host and pathogen proteins. High-throughput experimental screens have been primarily used to identify intraspecies PPIs [8–16]. However, recent efforts to include host-pathogen PPIs in public databases have made it easier to acquire the data needed to address these important questions.

In this paper, we integrate experimentally verified human-pathogen PPIs for 190 pathogen strains from seven public databases [17–23]. We partition the strains into 54 different pathogen groups, where each group is made up of taxonomically related strains. We analyze the intraspecies network of PPIs between the 1,233 unique human proteins spanned by the host-pathogen PPIs, and find that pathogens, both viral and bacterial, tend to interact with hubs (proteins with many interacting partners) and bottlenecks (proteins that are central to many paths in the network) in the human PPI network.

**Figure 1:**
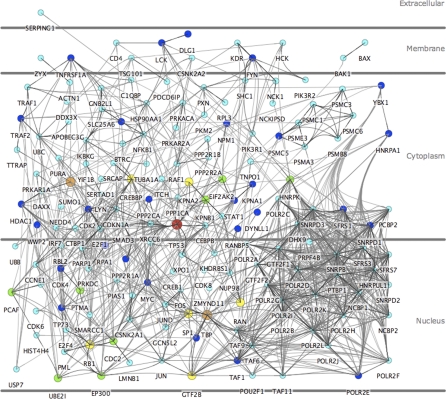
Human Proteins Interacting with Multiple Viral Pathogen Groups The network of interactions between human proteins interacting with at least two viral pathogen groups. The size and color of a protein denote the number of pathogen groups that interact with it: light blue is two, dark blue is three, green is four, yellow is five, orange is six, and red is seven.

**Figure 2:**
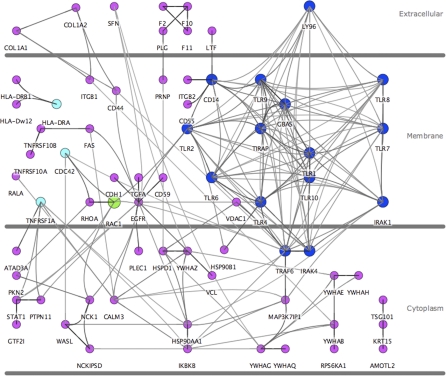
Human Proteins Interacting with Bacterial Pathogen Groups The network of interactions between human proteins interacting with at least one bacterial pathogen group. The size and color of a protein denote the number of pathogen groups that interact with it: purple is one, light blue is two, dark blue is three, and green is four.

We pay special attention to two networks of PPIs between human proteins: the proteins that interact with at least two viral pathogen groups (see Figure 1) and the proteins that interact with at least two bacterial pathogen groups (see Figure 2, noting that the figure also contains human proteins targeted by only one bacterial pathogen group). We used the Cerebral plugin [24] for Cytoscape [25] to render these images. We compute the Gene Ontology (GO) [26] functions enriched in each of these two sets of human proteins. Such enriched functions highlight human pathways that may be involved in infection mechanisms that are common to multiple pathogens. Examples of such processes and components include cell cycle regulation, I-κB kinase/NF-κB cascade, and the nuclear membrane. These functions shed light on a number of features shared by different pathogens: interacting with human transcription factors and key proteins that control the cell cycle; transport of genetic material through the nuclear membrane (in the case of viruses) to subvert the host's transcriptional machinery; triggering an immune response via toll-like receptors; and activation of NF-κB signaling. We discuss in detail the importance of these and other enriched functions, as well as the proteins they annotate and the pathogens they interact with. Overall, these results provide the first global view of aspects of human cellular processes that are controlled by and respond to pathogens.

Our results should be interpreted with caution since no single pathogen may target all the proteins and PPIs we analyze. In addition, data for bacterial pathogens are scarce. However, we suggest that piecing together targeted human proteins across multiple pathogens has the potential to provide insights into common molecular mechanisms of infection and proliferation used by different pathogens.

## Results/Discussion

We use the term “pathogen group” to refer to a set of pathogen strains that are closely related taxonomically, i.e., they all belong to the same genus, or, in the case of viruses, the same family. We partition the 190 strains into 54 pathogen groups: 35 viral, 17 bacterial, and two protozoan. Nearly all of the 10,477 human-pathogen PPIs we collect are for viral systems (98.3%), with the majority belonging to the human-HIV system (77.9%). These human-pathogen PPIs involve 1,233 unique human proteins, of which 1,109 are known to interact with at least one other human protein. Of these 1,233 human proteins, 221 interact with at least two pathogen groups (182 with more than one viral pathogen and 20 with more than one bacterial pathogen).

### Pathogens Target Protein Hubs and Bottlenecks

Researchers have argued that the degree distribution of PPI networks is scale-free and follows the power law, i.e., the fraction of proteins in the network interacting with *k* other proteins is proportional to *k* ^−γ^, for some γ greater than zero, typically between two and three [27,28]. One feature of such networks is that they are robust in the face of attacks on random nodes. For instance, the removal of random subsets of nodes increases the diameter of the network only gradually [29,30]. In this context, the diameter is defined as the average length of the shortest paths between all pairs of proteins. However, the selective removal of even a small number of nodes of high degree can dramatically change the topology of the network [29,30].

**Figure 3:**
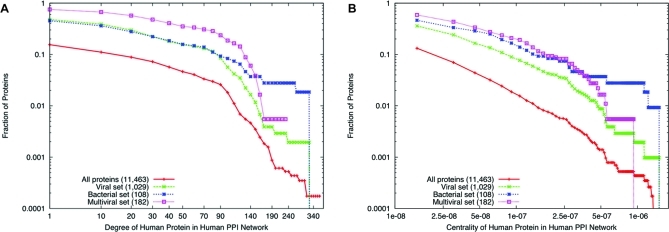
Degree and Centrality Distributions Cumulative log-log distributions of (A) node degrees and (B) centralities for four subsets of nodes in the human PPI network: (i) red pluses are the set of all proteins in the network; (ii) green squares correspond to the viral set; (iii) blue crosses are for the bacterial set, and (iv) magenta squares are for the multiviral set. Numbers in parentheses represent the number of proteins in each set. The fraction of proteins at a particular value of degree or centrality is the number of proteins having that value or greater divided by the number of proteins in the set.

There is considerable debate on the origins of the scale-free property and whether this property is an artifact of experimental biases and errors [31–33]. Notwithstanding this debate, we reasoned that pathogens may have evolved to interact with human proteins that are hubs (those involved in many interactions) or bottlenecks (those central to many pathways) [34] to disrupt key proteins in complexes and pathways. (See Methods for a precise definition of “bottleneck.”) Our results support this hypothesis. Figure 3A displays the cumulative log-log plot of the degree distribution of four sets of proteins in the human PPI network: (i) all proteins, (ii) “Viral” set, the subset of proteins interacting with at least one viral pathogen group, (iii) “Bacterial” set, the subset of proteins interacting with at least one bacterial pathogen group, and (iv) “Multiviral” set, the subset of proteins interacting with at least two viral pathogen groups. We did not include the “Multibacterial” set of human proteins interacting with two or more bacterial pathogen groups in this analysis since there are only 20 such proteins. These plots show that across almost the entire range of degrees, proteins interacting with viral and bacterial pathogen groups tend to have higher degrees than human proteins not interacting with pathogens. Further, proteins interacting with at least two viral pathogens have higher degrees than proteins interacting with one or more viral pathogens. The betweenness centrality results display the same trend (see Figure 3B). Across the entire range of values, proteins interacting with viral and bacterial pathogens have higher betweenness centrality. These results suggest that pathogens may have evolved to interact with human hub and bottleneck proteins, perhaps because these proteins control critical processes in the host cell.

We used Gene Set Enrichment Analysis (GSEA) [35] to test whether the gaps we observed in Figure 3 are statistically significant. GSEA is a method developed to assess the significance of the differential expression of a pre-defined gene set in two phenotypes of interest [35]. GSEA ranks all genes by a suitable measure of differential expression (e.g., the *t*-statistic) and uses a modified Kolmogorov-Smirnov test to assess if the genes in the given set have surprisingly high or low ranks. Since distributions of the *t*-statistics of differentially expressed genes have been observed to follow a power-law distribution [36], we reasoned that GSEA may be appropriate to test whether the human proteins interacting with pathogens have surprisingly high degree or betweenness centrality.

Our GSEA results support the conclusions we draw from Figure 3 that pathogens preferentially interact with human protein hubs and bottlenecks: for each of the three sets of proteins plotted in Figure 3, GSEA yields a *p*-value of at most 3 × 10^−5^ (degree) and 2.3 × 10^−4^ (centrality). To alleviate the concern that the observed patterns may be artifacts of experimental biases or errors in the human PPI network, we repeated each of the analyses using two subsets of the human PPI network: a network composed of 13,324 PPIs detected only by high-throughput studies [14,15,37] and a network with 59,396 PPIs constructed using only manually curated interactions [20,23]. The top half of Table 1 summarizes these results. For all three networks, the viral set, the bacterial set, and the multiviral set are significant at the 0.05 level for both degree and centrality, with the exception of the multiviral set in the high-throughput network. Since 77.9% of the human-pathogen PPIs are for the human-HIV system, we repeated these analyses for each network after removing all human-HIV PPIs and obtained similar results (see the bottom half of Table 1). In Text S1, we discuss three analyses that show that the consistency in the GSEA results for degree and for centrality are unlikely to result from any correlation that may exist between a protein's degree and its centrality (Figure S1 and Table S1 accompany the discussion in Text S1). We note that Tables S2 and S3 of the supplementary data contain detailed information on the GSEA results for the groups in Figure 3 and for individual pathogen groups.

**Table 1:**
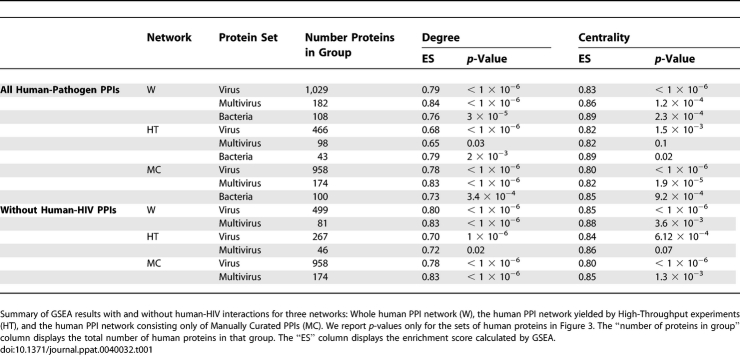
GSEA Results

### Functions Enriched in Proteins Interacting with Pathogens

We computed over-represented GO terms in 58 sets of human proteins: the bacterial set, the viral set, the multibacterial set, the multiviral set, and the 54 sets of human proteins interacting with each of the 54 pathogen groups. Overall, we found 404 unique GO terms enriched in these sets. A complete list of enriched GO terms with images of the sub-networks spanned by the human proteins annotated with each term is available on the supplementary website.

We identified at least one enriched function in 21 pathogen groups. Analysis of these data identified 91 biclusters (see Methods for details), each containing between two and seven pathogen groups and between two and 40 enriched GO functions. We focus on two of the biclusters below. The biclusters demonstrate that our analysis can group different enriched functions together even if the effects of the interactions on the host cell or the participating host proteins are different.

Our first example is a bicluster spanning the three pathogen groups Adenovirus, HIV, and Papillomavirus and 23 GO functions. GO biological processes in the bicluster include “cell cycle process” and “regulation of cellular process.” GO cellular components in the bicluster include “membrane-enclosed lumen” and “pore complex.” The membrane-enclosed lumen is the space within a sealed membrane or between two sealed membranes. Proteins annotated with these functions include KPNA2, a karyopherin, the histone deacetylases HDAC1 and HDAC2, and a number of Transcription Factors (TFs). KPNA2 plays an important role in both the import and export of material through the nuclear membrane. Interactions with KPNA2 enable a virus to enter the nucleus and take over the host's transcriptional machinery [38–41]. HDACs play an important role in silencing gene expression by removing acetyl groups from histones, thus causing them to wrap more tightly around DNA and block the binding of TFs. The role played by pathogen-HDAC interactions varies among pathogen groups. In the case of Adenovirus, it has been suggested that the pathogen protein E1B interacts with HDAC1/SIN3 to produce an enzymatically active complex that may be capable of repressing the transcriptional activity of the human TP53 protein in order to block apoptosis [42]. In contrast, the E7 Papillomavirus protein binds to the HDAC complex to promote cell growth, eventually leading to cervical cancer [43].

The second example is a bicluster containing a virus (HIV) and three bacteria (Chlamydia, Neisseria, and Escherichia coli). This bicluster contains 11 GO functions including the biological processes “immune response,” “response to stimulus,” and “cytokine production.” Although these four groups of pathogens interact with proteins belonging to the same pathways, the functions of the interactions are different. In the case of the bacteria, these functions annotate such proteins as toll-like receptors (TLRs) and interleukin receptor-associated kinases (IRAKs), which are special classes of host proteins responsible for recognizing foreign material and activating an immune response. There are no reported interactions with these proteins and HIV, although some researchers suggest that the single-stranded RNA of HIV-1 may encode many TLR7/TLR8 ligands [44]. In contrast to the bacteria in the bicluster, HIV uses host proteins involved in immune response such as CD4, CCR5, and CXCR4 to gain entrance to the cell. HIV attaches to the host protein CD4, a T cell glycoprotein, and subsequently to host chemokine receptors CCR5 and CXCR4. These binding events cause conformational changes to host proteins that allow the membrane of the virus to fuse to the host cell membrane [1].

### The Network of Proteins Interacting with Multiple Pathogens

The biclustering analysis of the previous section suggests that specific sets of pathogen groups might trigger or target the same human pathways and processes. Encouraged by these data, we asked if there are infection pathways commonly targeted or triggered by at least two viral or bacterial pathogen groups. To answer this question, we constructed two networks of human proteins: one where every protein interacts with at least two viral pathogen groups and the other where every protein interacts with at least two bacterial pathogen groups. In each network, we included every PPI connecting two proteins in the network. Figures 1 and 2 display these networks. (Note that Figure 2 also contains human proteins that interact with only one bacterial pathogen group.) We computed the enriched GO functions in these two networks. We group and highlight some of the enriched functions and relevant sub-networks below. Throughout our discussion, we will refer to the localization of proteins in the four main regions of Figures 1 and 2: extracellular, the cell membrane, the cytoplasm, and the nucleus. For every GO function that we discuss, we mention its *p*-value and rank in the sorted list of all functions enriched in the corresponding network.

### Human Proteins Targeted by Multiple Viral Pathogens

Our analysis highlights a number of important mechanisms that viral pathogens use to manipulate the human cell: (i) control the host cell cycle program to ensure the transcription of viral genetic material; (ii) utilize human TFs to promote the transcription of viral genetic material; (iii) target key human proteins that regulate critical cellular processes such as apoptosis; and (iv) subvert host machinery for transporting material across the nuclear membrane.

#### Control the host cell cycle program.

**Figure 4:**
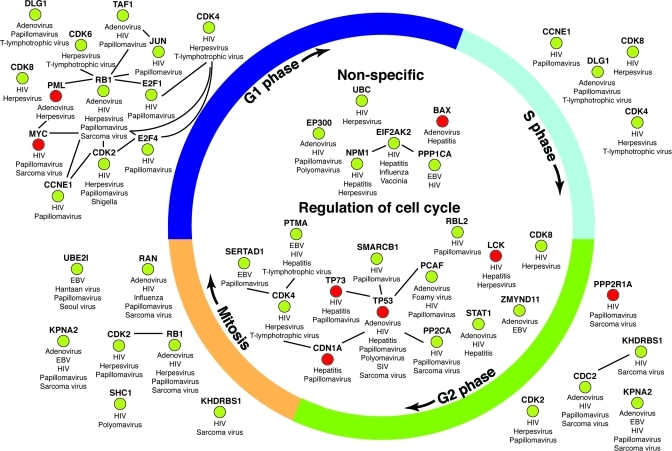
Human Cell Cycle Proteins Interacting with Multiple Viral Pathogen Groups Enriched network of human proteins annotated with “cell cycle.” The subset of proteins labeled as “Non-specific” are those not annotated with any function more specific than “cell cycle” in GO. If a protein participates in multiple phases, then it appears in each phase. An edge connecting two proteins denotes a known interaction in the human PPI network. Human proteins highlighted in red are those known to be involved in the induction of apoptosis.

Many viral pathogens are known to manipulate host cell cycle processes [45–47]. Our enrichment results reflect these findings. Our analysis identifies a sub-network of human proteins targeted by multiple viral pathogen groups enriched in the biological process “cell cycle” (*p*-value 6.2 × 10^−6^, rank 21/89). Figure 4 displays this network. In this figure, we used GO annotations to clarify in which phase of the cell cycle each protein participates. The proteins in this figure are scattered through the cytoplasm and nucleus regions of Figure 1.

Two stages of the cell cycle are enriched in our analysis: “G_1_ phase” (*p*-value 0.004, rank 52/89) and “Interphase” (*p*-value 0.01, rank 60/89). Images for these functions are available on the supplementary website. G_1_ is the initial stage of the cell cycle. In this phase, a number of proteins needed for DNA replication are transcribed and translated. A direct link between pathogen interference and the G_1_ phase has been established for HIV [48]. The HIV TAT protein elongates the G_1_ phase in order to promote viral gene expression. Of the 13 human proteins in Figure 4 that participate in G_1_, ten are known to interact with TAT. One of these interactions is with the human protein RB1, a retinoblastoma-associated protein and a known tumor suppressor, which can repress genes transcribed by the E2F family of transcription factors that are required for entering the S phase of the cell cycle [49]. RB1 interacts with five pathogens in total: Adenovirus, Herpesvirus, HIV, Papillomavirus, and Simian virus [50–54]. In the case of HIV, the TAT protein interacts with the human RB1 protein to manipulate normal cell cycle conditions and promote viral gene expression. The HIV long terminal repeat (LTR) is responsible for integrating viral DNA into the host genome and also acts as a promoter and enhancer of viral proteins. The LTR is most active in the early G_1_ phase and the activity of the LTR diminishes as the cell progresses through the G_1_ phase and enters the S phase [48]. Therefore, the extension of the G_1_ phase may increase activity of the LTR and the eventual production of more viral proteins. In the case of Papillomavirus, the VE6 protein in Papillomavirus has been shown to manipulate the cell cycle by altering mitotic checkpoint fidelity through its effect on CDC2 activity and inactivation of TP53 [55]; it interacts with ten human proteins in Figure 4.

The human DLG1 protein is a “discs large homolog” that is essential for the transition from the G_1_ to S phase of the cell cycle. This protein interacts with three pathogens: Adenovirus, Papillomavirus, and T-lymphotrophic virus [56,57]. The direct interaction of Papillomavirus proteins with human DLG1 has been implicated in development of HPV-related cancer [58].

Our analysis also identifies a network of human proteins enriched with the GO function “transcription regulator activity” (*p*-value 3.22 × 10^−7^, rank 15/89) (see supplementary website for image). The portion of Figure 4 corresponding to the G_1_ phase includes the transcription factors E2F1, E2F4, and TAF1. Each of these proteins plays a key role in normal cell cycle progression from G_1_ to S phase. E2F1 and E2F2 interact with two pathogens, HIV and Papillomavirus [48,59,60]. TAF1 interacts with three pathogens, Adenovirus, HIV, and Papillomavirus [61–63]. By blocking the interaction of RB1 and various transcription factors, viral pathogens are able to prevent the cell from advancing into the S phase. This event extends the G_1_ phase of the cell cycle and allows the transcription of viral genetic material.

#### Regulate apoptosis.

An important step in viral pathogenesis is the regulation of host cell apoptosis. During the initial process of infection, prevention of apoptosis is important to allow the replication of viral genetic material. However, promotion of apoptosis has been implicated in the progression of infection. Our results underscore both phenomena. Several host proteins involved in the control of cellular apoptosis are targeted by viral pathogens (human proteins highlighted in red in Figure 4). One of the key regulators of apoptosis, and perhaps the most studied human protein, is TP53. TP53 interacts with seven viral pathogens: Adenovirus, Hepatitis, HIV, Papillomavirus, Polyomavirus, Sarcoma virus, and Simian virus [20, 64–70]. Interactions with Adenovirus, Hepatitis, and Papillomavirus are responsible for preventing apoptosis of the infected human cell. Adenovirus E1B and E4 proteins bind with and inactivate TP53 [71,72]. The human Survivin protein is an apoptosis inhibitor that is repressed by TP53 [73]. The repression of Survivin is necessary for the human cell to activate apoptotic programming. Another study shows that the HIV VPR protein can directly upregulate the human Survivin protein [74]. These studies suggest a common mechanism for viral inhibition of apoptosis of the host cell. TP53 interacts with a number of Hepatitis proteins including the Core protein; Core has been shown to augment TP53′s transcriptional activity during infection to promote production of viral proteins and deregulate cell cycle checkpoint controls and block TP53-mediated apoptosis [75,76]. Papillomavirus VE6 interacts with human TP53 to promote degradation of TP53 and prevent apoptotic programming of the infected cell [77]. In contrast to these phenomena, the viral HIV protein TAT has been shown to assist in the progression of HIV infection by attaching to uninfected host T cells and triggering cell death via apoptosis [78,79].

#### Transport viral material across the nuclear membrane.

**Figure 5:**
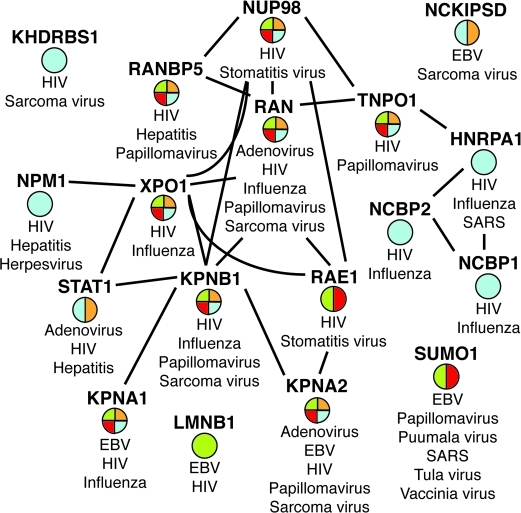
Human Nuclear Membrane Proteins Interacting with Multiple Viral Pathogen Groups Enriched network of human proteins annotated with “nuclear transport” (blue), “nuclear membrane part” (green), “protein import” (orange), and “nuclear pore” (red). An edge connecting two proteins denotes a known interaction in the human PPI network.

Since viruses lack the machinery needed to replicate their genomes, viral genetic material must first cross the barrier from the cytoplasm into the nucleus in order to make use of the host's transcriptional machinery. Our analysis identifies a subset of human proteins enriched in four GO functions related to this important step: “nuclear transport” (*p*-value 2.32 × 10^−5^, rank 24/89), “nuclear membrane part” (*p*-value 5.61 × 10^−5^, rank 28/89), “protein import” (*p*-value 0.001, rank 41/89), and “nuclear pore” (*p*-value 0.018, rank 69/89). Figure 5 displays this network. The layout in Figure 1 displays these proteins both in the region labeled “cytoplasm” and in the region labeled “nucleus.”

The nuclear pore is a large protein complex that spans the nuclear membrane and allows for the transport of molecules across the nuclear envelope including proteins and RNA. There are ten human proteins that are part of the nuclear pore and targeted by multiple pathogens. These are the nodes containing a red section in Figure 5. Although smaller molecules may freely pass through the nuclear pores of the nuclear envelope, larger macromolecules require the assistance of karyopherins. Karyopherins may act as importins or exportins. Karyopherins bind to their cargo; after they cross the nuclear envelope, an interaction with the human RAN protein releases the bound partner. Figure 5 contains five human karyopherin proteins (KPNA1, KPNA2, KPNB1, RANBP5, TNPO1) as well as the human RAN protein, which interacts with five pathogens: Adenovirus, HIV, Influenza, Papillomavirus, and Sarcoma virus [20,80]. The human protein KPNB1 interacts with four pathogens: HIV, Papillomavirus, Influenza, and Simian virus [20,39,81,82]. In the case of HIV, one of the interacting partners of the human KPNB1 protein is REV. KPNB1 binds and mediates the nuclear import of the HIV REV protein. Once inside the nucleus, REV binds to unspliced viral mRNA and exports it from the nucleus to be translated [6]. REV is able to move between the nucleus and cytoplasm because it contains both a nuclear localization signal and a nuclear export signal. The human RANBP5 protein interacts with three pathogens: HIV, Hepatitis, and Papillomavirus [83–85]. The Hepatitis interactor for RANBP5 is the viral 5A protein. While little is known about the RANBP5 protein, studies suggest that the viral 5A protein may interact with RANBP5 and block secretion of cytokines produced in response to a viral infection [83]. This network highlights the ability of viral pathogens to make use of host machinery in order to translate their own genetic material and at the same time prevent the activation of a viral immune response.

### Human Proteins Targeted by Multiple Bacterial Pathogens

**Figure 6:**
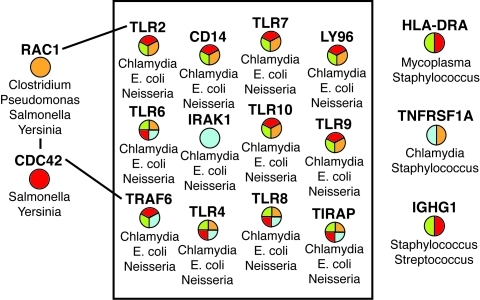
Human Immune System Proteins Interacting with Multiple Bacterial Pathogen Groups Enriched network of human proteins annotated with “immune system process” (red), “response to wounding” (orange), “immune response” (green), and “I-κB kinase/NF-κB cascade” (blue). The proteins in the black box form a dense network of PPIs; we have left these out for clarity. An edge connecting two proteins denotes a known interaction in the human PPI network.

Although the number of human-bacteria PPIs gathered in this study is small (only 174), our methods identified an important subset of human proteins enriched for functions involved in immune response and interacting with multiple bacterial pathogen groups. Figure 6 displays a subset of the multibacterial set that is enriched in four GO functions: “immune system process” (*p*-value 1.397 × 10^−9^, rank 1/28), “response to wounding” (*p*-value 3.93 × 10^−4^, rank 8/28), “immune response” (*p*-value 0.002, rank 14/28), and “I-κB kinase/NF-κB cascade” (*p*-value 0.012, rank 18/28). The proteins contained in this image are located in the top-right corner of Figure 2.

These functions are tied together by the Toll-Like Receptors (TLRs) and the protein IRAK1 found in the network in Figure 6. TLRs are a special class of cell-surface proteins that play a role in recognizing the presence of a pathogen and activating an immune response against the pathogen. The TLR/IRAK complex stimulates the activity of NF-κB [86–88], a complex of proteins that act as a TF for activating the production of a set of proteins in response to stimuli such as stress, cytokines, and bacterial or viral antigens.

The human TLRs and IRAK1 protein interact with the pathogen proteins FLIC (E. coli), HSP60 (Chlamydia), and PIB (Neisseria) [20]. FLIC is a flagellin protein. TLR4 and TLR5 contain a specific innate immune receptor for recognizing bacterial flagella [5,89]. HSP60 is a heat-shock protein that stimulates an immune response via TLR2 and TLR4 [90]. PIB is an outer membrane protein that is known to be recognized by TLR2, TLR4, and TLR9 [7].

Another human protein included in this network is HLA-DRA, which is part of the major histocompatibility complex (MHC). The MHC plays an important role in the immune system. HLA-DRA belongs to the class II MHC; proteins in this class belong to the lysosomal compartment of the cell, which contains digestive enzymes that kill engulfed foreign particles such as viruses or bacteria. The two bacterial partners for HLA-DRA are Mycoplasma and Staphylococcus [91,92]. In the case of Mycoplasma, the interacting partner is the MAM superantigen, which is known to contribute to autoimmune disease by activating proinflammatory monokines such as interleukin 1β and the tumor necrosis factor α [93].

### Other Highly Targeted Human Proteins

The networks in Figures 1 and 2 contain a number of other human proteins targeted by more than two pathogen groups. We discuss two of these proteins—STAT1 and EP300.

Viral pathogens also interact with other human proteins involved in immune response pathways that are not included in the network in Figure 6. An example is the human protein STAT1. When the cell recognizes the presence of foreign material, it activates an immune response as a defense mechanism to either remove the foreign material or cause the cell to undergo apoptosis. During this process, STAT1 is tyrosine- and serine-phosphorylated and forms a homodimer known as IFN-γ-activated factor (GAF). GAF migrates to the nucleus where it binds to specific *cis*-elements to drive the cell to produce interferons, agents that inhibit viral replication within other cells of the body [94]. STAT1 interacts with Adenovirus, HIV, and Hepatitis [95–97]. Hepatitis POLG is part of the pathogen core complex that allows the virus to avert host antiviral response by binding to host STAT1 and inhibiting its activity [98].

Within the nucleus, we see pathogens target the human protein EP300, a histone acetyltrans-ferase that regulates transcription via chromatin remodeling. EP300 interacts with Adenovirus, HIV, Papillomavirus, and Polyomavirus [99–102]. The pathogen Adenovirus targets human EP300 via E1A. E1A is an oncoprotein that stimulates cell growth and inhibits differentiation by binding to the EP300/CBP complex and deregulating cellular transcription programs [103]. Papillomavirus protein VE7 shares many functional and structural similarities with E1A and is an interacting partner of human EP300. The disruption of normal growth conditions brought about by the E1A-EP300 interaction leads to the development of cervical cancer [104]. In the case of HIV, the viral TAT protein targets human EP300. The resulting complex regulates TAT transactivating activity and may assist in the integration of viral genetic material into human DNA [105].

### Conclusions

We have provided a general overview of the landscape of human proteins interacting with pathogens and demonstrated that pathogens preferentially interact with two classes of human proteins: hubs (i.e., proteins that interact with many other human proteins) and bottlenecks (i.e., proteins that lie on many shortest paths) in the human PPI network. We identified GO functions over-represented in human proteins interacting with pathogens. Biclustering analysis demonstrated that many sets of pathogen groups target the same processes in the human cell, even if they interact with different proteins.

We constructed networks of PPIs between human proteins that interact with at least two viral pathogen groups and with at least two bacterial pathogen groups. Consideration of the GO functions enriched in these networks provided insights into numerous pathways targeted or triggered by multiple pathogens: control and deregulation of the cell cycle; import of pathogen proteins into the nucleus in an attempt to subvert the host's DNA replication and transcription machinery; manipulation of host cellular programs such as apoptosis; immune response and activation of NF-κB pathways via the TLR/IRAK complex.

A striking aspect of this network is that human proteins that mediate pathogen effects are often proteins in cancer pathways (e.g., RB1, TP53, and STAT1). We note that only some of the pathogens targeting such proteins are known to cause cancer themselves (e.g., Herpesvirus and Papillomavirus). In fact, a number of parallels are becoming evident between infection and cancer; for instance, in the part that TLRs play in angiogenesis and their potential as targets for therapeutics [106,107] and the role that viruses may play in the development of inflammatory diseases and cancer [108]. Cell cycle regulators and many TFs have been extensively studied in the context of mediating tumor formation. Our observation that they are also communication vehicles for pathogens suggests that the link between pathogen infection and cancer may be worthy of further experimental studies.

An important outcome of such a comparative study is the identification of human proteins to target experimentally for developing therapeutics. We provide a file on the supplementary website that contains the degree, centrality, the number of pathogen interactors, and the most specific annotations in each of the three GO hierarchies for each human protein that interacts with at least one pathogen protein. We provide this data as a resource for researchers interested in prioritizing antiviral and antibacterial targets.

We reiterate that our results should be interpreted with caution since no single pathogen may target all the proteins we analyze. As interactions between host and pathogen molecules are discovered on genome-wide scales [109], computational analyses such as those presented in this paper may provide a more detailed understanding of the landscape of host pathways and processes that pathogens target.

## Methods

### Datasets used.

We downloaded all datasets used in this study in August 2007. We gathered 10,477 experimentally detected and manually curated protein-protein interactions (PPIs) between human and pathogen proteins and 75,457 experimentally verified PPIs between human proteins from primary literature [109] and seven databases: the Biomolecular Interaction Network Database [21], the Database of Interacting Proteins [19], the Human Protein Reference Database [23], IntAct [18], the Molecular INTeraction database [17], the Munich Information Center for Protein Sequences [22], and Reactome [20]. Table 2 contains statistics on the experimental methods that yielded these PPIs and the literature support for the PPIs. These interactions cover 190 different pathogen strains. Two pathogens—HIV and Hepatitis—account for 88.4% (9,268) of the human-pathogen PPIs. To mitigate this bias, we merged pathogen strains into 54 groups based on taxonomic similarity: each group contains pathogens belonging to the same genus, or, in the case of viruses, the same family. The 54 pathogen groups contain 35 viral, 17 bacterial, and two protozoan groups. We constructed lists of unique human proteins interacting with each group. Table 3 summarizes the number of interactions acquired for each pathogen group. For some analyses, we consider a human PPI network assembled from unbiased high-throughput experiments [14,15,37] and a network constructed from only manually curated human PPIs [20,23]. These networks contain 13,324 and 59,396 interactions, respectively. We obtained functional annotations from the Gene Ontology (GO) [26].

**Table 2:**
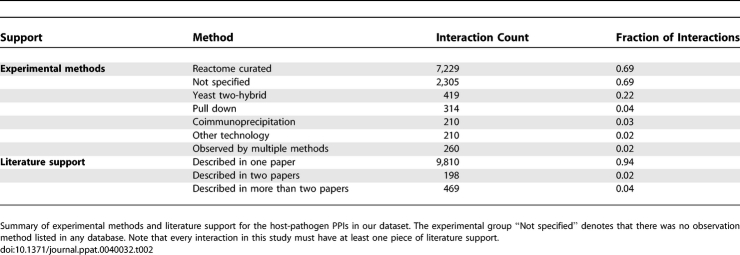
Interaction Method and Support Summary

**Table 3:**
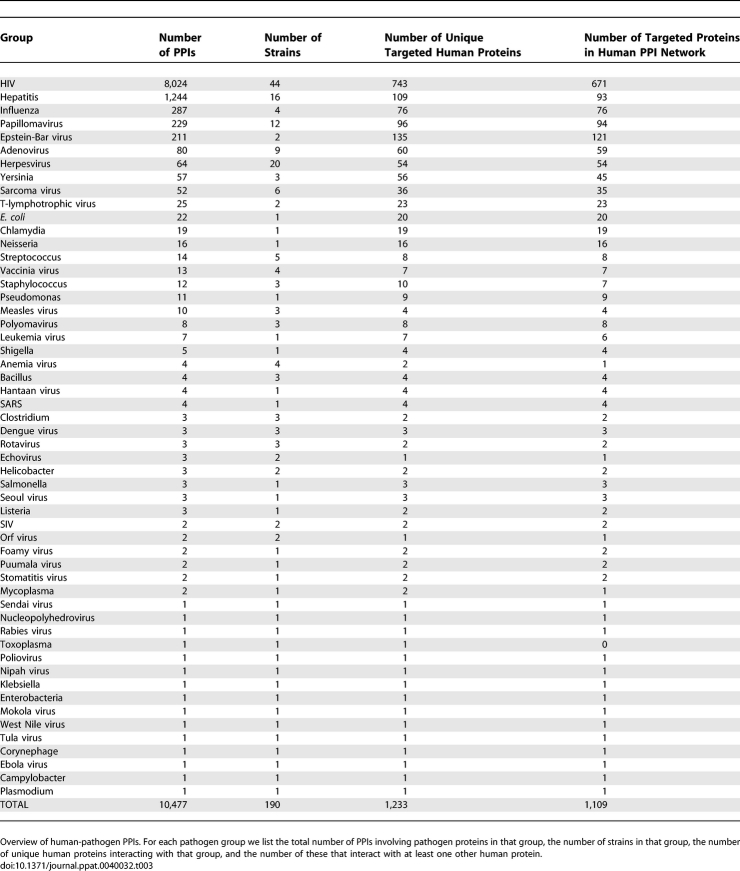
Interaction Summary

### Notation.

We represent the set of known interactions between human proteins as an undirected graph *G(V, E)*, where *V* is the set of nodes (proteins) and *E* is the set of edges (interactions). Let *M* be the set of pathogen groups. We say that a pathogen group *P* interacts with a human protein *s* if *s* interacts with a protein in *P*. For a pathogen group *P* ∈ *M*, we define V_P _⊆ V to be the set of human proteins that interact with *P*. Let *T* = ∪*_P_*_∈*M*_ be the set of proteins that interact with at least one pathogen. Let *T_V_* (respectively, *T_B_*) be the set of human proteins that interact with at least one viral (respectively, one bacterial) group. Let *T^(k)^_V_* ⊆ *T_V_* (respectively, *T^(k)^_B_* ⊆ *T_B_*) be the set of human proteins that interact with at least *k* viral (respectively, k bacterial) pathogen groups; by definition, *T^(1)^_V_* ≡ *T_V_* and *T^(1)^_B_* ≡ *T_B_*. We now describe in detail the tests we use to analyze *T_B_*, *T_V_*, *T^(2)^_B_*, *T^(2)^_V_*, and the 54 *V_P_* sets.

### Analysis of degree in the human PPI network.

The *degree* of a protein in a graph is the number of interactions in which it participates, not including self-interactions. We plot distributions of the degrees of four sets of proteins in *G*: (i) *V*, the set of all proteins in *G*; (ii) *T_B_*, the set of all human proteins interacting with at least one bacterial pathogen group; (iii) *T_V_*, the set of all human proteins interacting with at least one viral pathogen group; and (iv) *T^(2)^_V_*, the set of human proteins interacting with at least two viral pathogen groups. In this analysis, we ignore *T^(2)^_B_* since it contains only 20 proteins. If the distributions of *T_B_* and *T_V_* are more biased towards high degree proteins than the distribution for *V*, then we hypothesize that viral and bacterial pathogens have evolved to interact with hub proteins in the human PPI network.

### Analysis of betweenness centrality in the human PPI network.

The degree of a protein captures only its local connectivity. Centrality captures both global and local features of a protein's importance in a network. In this paper, we use the notion of a protein's betweenness centrality [110]. A protein with high betweenness centrality is characteristic of a bottleneck in an interaction network (i.e., there are many paths that pass through this protein) [34].

We define the *betweenness centrality bc(v)* of a protein *v* as the fraction of shortest paths in *G* between all protein pairs (*u,w*) that pass through the protein *v*. Given *u*, *v*, *w* ∈ *V*, let σ*_uw_* denote the number of shortest paths between proteins *u* and *w.* There may be multiple equally long paths between *u* and *w* that are shorter than any other path between *u* and *w.* Let σ*_uw_*(*v*) denote the number of these that pass through *v*. Then the betweenness centrality of *v* is

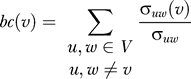



In our analysis, we divide *bc*(*v*) by the number of pairs of nodes in *G*, yielding a quantity between 0 and 1. We use the algorithm devised by Brandes [111] to compute the betweenness centrality of all nodes in *G*. This algorithm runs in time proportional to the product of the number of nodes in *G* and the number of edges in *G*. As with the degree analysis, we plot distributions of the betweenness centrality for *V*, *T_B_*, *T_V_*, and *T^(2)^_V_*. If the distributions for *T_B_*, *T_V_*, and *T^(2)^_V_* are biased toward higher values of centrality than the distribution for *V*, we hypothesize that pathogens have evolved to interact with bottlenecks in the human PPI network.

### Gene set enrichment analysis.

Let *L* be the ranked list of the proteins in *V*, where we rank the proteins either by degree or by betweenness centrality. Given *L* and a predefined set *S* of proteins of interest (e.g., those interacting with HIV), we use GSEA to determine whether the proteins contained in *S* are randomly distributed throughout *L* or concentrated at the top. In the ranked list *L*, let *l_i_* be the value (of degree or centrality) at index *i*; 1 ≤ *i* ≤ |*L*|. We abuse notation and say that an index *i* is an element of *S* if the protein whose rank is *i* belongs to *S*. First, we compute *m* = Σ*_i_*_∈*L*_ *l_i_*, the sum of all the values in *L*. Next, for each index *i* in *L*, we compute two values:

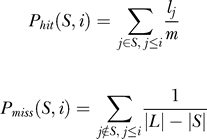



**Table 4:**
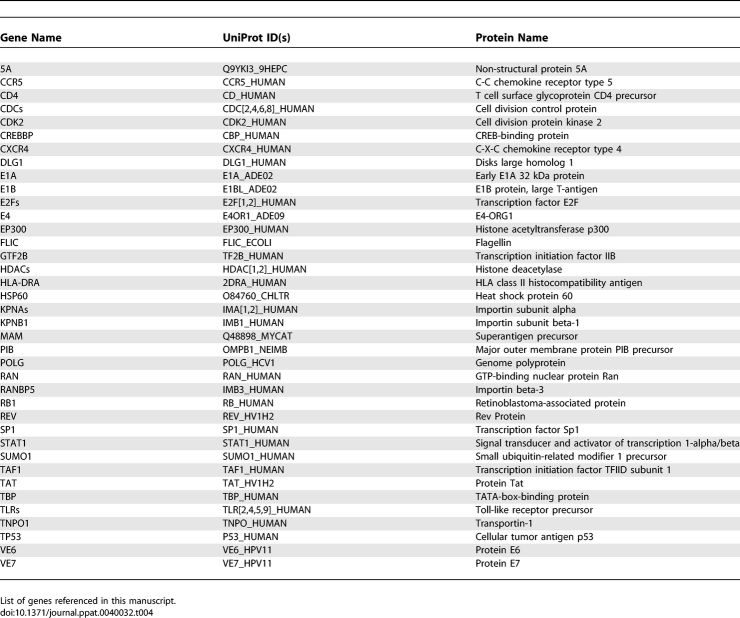
Gene List

Thus, *P*_*hit*_(*S*, *i*) measures the weighted fraction of proteins with index at most *i* that are in *S* and *P*_*miss*_(*S*, *i*) measures the fraction of proteins with index at most *i* that are not in *S*. We handle multiple ranks with identical values by computing these two values only at the largest rank for each unique value in *L*. Finally, we define the enrichment score as the largest positive value of *P*_*hit*_(*S*, *i*) - *P*_*miss*_(*S*, *i*), i.e.,

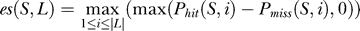



A large positive value of *es*(*S*, *L*) indicates that the proteins in *S* have high degree or high betweenness centrality. Note that our modification of the original definition of the enrichment score [35] ensures that if *S* mainly contains proteins with low degree or betweenness centrality, then the score will be close to 0, since *P*_hit_(*S*, *i*) − *P*_miss_(*S*, *i*) will be negative for most indices. We record the rank *i* that yields *es*(*S*, *L*); the column titled “#proteins contributing” in Table S1 of the supplementary data displays these numbers. To compute *p*-values for an observed enrichment scores, we generate a null distribution of scores by repeatedly selecting |*S*| random nodes in *L* and computing the score for each random subset of nodes. We repeat this process 1,000,000 times and estimate the *p*-value for *s* as the fraction of random sets whose score is at least as large as *s*. We obtain our results by testing each of 57 sets: *T_B_*, *T_V_*, *T^(2)^_V_*, and the sets *V_P_* corresponding to each of the 54 pathogen groups.

### Functional enrichment.

We isolate functionally coherent subsets of human proteins among the sets *T_B_*, *T_V_*, *T^(2)^_B_*, *T^(2)^_V_*, and the sets *V_P_* corresponding to each of the 54 pathogen groups using a test for functional enrichment. Given the hierarchical structure of the Gene Ontology (GO) [26], we account for dependencies between annotations by using the method proposed by Grossman et al. [112]. Let *S* be a set of proteins of interest (e.g., the set of proteins interacting with HIV). We aim to compute GO functions that annotate a surprisingly large number of proteins in *S*. To this end, for each function *f* in GO, we count *s_f_*, the number of proteins in *S* annotated with *f* and *s_pa_*_(*f*)_, the number of proteins in *S* annotated by at least one parent of *f*. We also compute *v_f_* and *v_pa_*_(*f*)_, the number of proteins in *V* annotated by *f* and by at least one parent of *f*, respectively. With these four counts in hand, we use the hypergeometric distribution to compute the probability *p_f_*(*S*, *V*) of drawing *s_f_* or more proteins from a set of *v_f_* marked proteins when we select *s_pa_*_(*f*)_ proteins at random from a universe of *v_pa_*_(*f*)_ proteins:

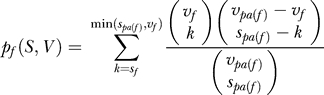



We account for multiple hypothesis testing using the method of Benjamini and Hochberg [113]. We consider only functions enriched with a *p*-value of at most 0.05. Note that different enriched functions may annotate identical sets of human proteins. In each such case, we group the functions and associate the most enriched function (and its *p*-value) with the group. To report enrichment ranks, we sort the groups in increasing order of *p*-value. Although not discussed in this paper we repeat this analysis using *T* (rather than *V*) as the universe of proteins. With *T* as the universe, we expect to find functions that distinguish between the pathogens. The results with *T* as universe are available on our supplementary website.

### Biclustering of enriched functions.

We compute enriched functions in each of the 54 sets of human proteins interacting with each pathogen group. We construct a binary matrix whose rows are enriched functions and whose columns are pathogen groups. An entry is one in this matrix if and only if the function is enriched with a *p*-value of at most 0.05 in the pathogen. In this binary matrix, we define a bicluster to be a subset *R* of rows and a subset *C* of columns such that each row-column pair in *R* × *C* contains a one. We also require a bicluster to be closed, i.e., each row not in *R* (respectively, column not in *C*) contains a zero in at least one column in *C* (respectively, row in *R*). We use the Bimax algorithm to compute all closed biclusters in this binary matrix [114].

## Supporting Information

Figure S1 Protein Degree–Centrality Scatter-Plots Log-log scatter-plots of each protein contained within the three networks used in this study: (A) the whole human PPI network (11,463 proteins), (B) the high-throughput human PPI network (4,986 proteins), and (C) the manually curated human PPI network (8,704 proteins). The *x*-axis is the degree and the *y*-axis is the centrality of a protein within its respective network.
(2.5 MB TIF)

Table S1 Relative Node Occurrences Relative occurrences of four types of nodes in each of the three networks: Whole human PPI network (W), the human PPI network yielded by High-Throughput experiments (HT), and the human PPI network consisting only of Manually Curated PPIs (MC). The “Fraction” column defines the cutoff at which a protein is considered a hub or a bottleneck. The other columns represent the fraction of hub-bottleneck, non-hub-bottleneck, hub-non-bottleneck, and non-hub-non-bottleneck proteins in the network using that cutoff.
(46 KB PDF)

Table S2 Detailed GSEA Results Summary of GSEA results with and without human-HIV interactions for three networks: Whole human PPI network (W), the human PPI network yielded by High-Throughput experiments (HT), and the human PPI network consisting only of Manually Curated PPIs (MC). We report *p*-values only for the sets of human proteins in Figure 3. The “#proteins in group” column displays the total number of human proteins in that group. The “ES” column displays the enrichment score calculated by GSEA. The column titled “#proteins contributing” displays the number of proteins contributing to the ES score (see Methods for details.) The column titled “Jaccard's” lists the Jaccard coefficient between the two sets of proteins contributing to the ES score for degree and for centrality.
(67 KB PDF)

Table S3 Detailed GSEA Results for Individual Pathogen Groups Summary of GSEA results for individual pathogen groups for three networks: Whole human PPI network (W), the human PPI network yielded by High-Throughput experiments (HT), and the human PPI network consisting only of Manually Curated PPIs (MC). We report *p*-values only for the sets of human proteins in Figure 3. The “#proteins in group” column displays the total number of human proteins in that group. The “ES” column displays the enrichment score calculated by GSEA. The column titled “#proteins contributing” displays the number of proteins contributing to the ES score (see Methods for details.) The column titled “Jaccard's” lists the Jaccard coefficient between the two sets of proteins contributing to the ES score for degree and for centrality. We only report groups that are enriched for both degree and centrality.
(72 KB PDF)

Text S1 Supporting Text Testing correlation between protein degree and protein centrality for GSEA analysis and GSEA analysis for individual pathogen groups.
(59 KB PDF)

### Accession Numbers

Table 4 contains a list of all the proteins discussed in this paper and their corresponding UniProt ids and descriptions.
