# Supplementary material for: The Landscape of Human Proteins Interacting with Viruses and Other Pathogens
Source: PLoS Pathog. 2008 Feb 15;4(2):e32. doi: 10.1371/journal.ppat.0040032 (PMC2242834; doi:10.1371/journal.ppat.0040032)
Supplement: Supplementary file 1 [file ppat.0040032.sd001.pdf]

# The Landscape of Human Proteins Interacting with Viruses and Other Pathogens (Supplementary Data)

## 1 Testing correlation between degree and centrality

The GSEA results for degree and centrality appear to be consistent with each other. We per-formed three analyses to discount the possibility that this correlation arises from an existing cor-relation between a human protein's degree and its centrality. We applied these analyses to three networks: the whole human network (W), the human PPI network yielded by High-Throughput experiments (HT), and the human PPI network consisting only of Manually Curated PPIs (MC).

1. We constructed scatter plots of each protein's degree and centrality. As shown in Supplementary Figure S1, these plots show that there is significant de-correlation between these two quantities. In particular, there are many hub-non-bottleneck (top-left portion of each plot) and non-hub-bottleneck proteins (bottom-right portion of each plot). Similar findings of non-hub-bottleneck and hub-non-bottleneck proteins have been found in networks of other model organisms [1].

2. Yu et al. [1] classify a protein as a hub or bottleneck by first sorting proteins according to the degree (respectively, centrality) and by classifying proteins with the top 20% of degree values (respectively, centrality values) as hubs (respectively, bottlenecks). Using different cutoffs of classification (10%, 20%, 30%, and 40%) of a protein as a hub or as a bottleneck, we calculated the fraction of hub-bottleneck, non-hub-bottleneck, hub-non-bottleneck, and non-hub-non-bottleneck proteins contained in each of the three networks. Supplementary Table S1 shows that in each network, there are a non-negligible number of nonhub-bottleneck proteins.

3. For each GSEA analysis, we determined the set of proteins that contribute to the ES score (see Methods for details). For each pathogen group and for the virus set, the bacteria set, and the multivirus set, we computed the Jaccard's coefficient of the set of proteins that contributed to the degree ES score and the set that contributed to the centrality ES score. Supplementary Table S2 is a copy of Table 1 in the main paper, except for additional columns listing the number of proteins contributing to the ES score (columns 5 and 8) and the Jaccard's coefficient of these two sets (the last column). As the last column indicates, the Jaccard's coefficient ranges from 0.45 to 0.7, which suggests that while there is some overlap between the sets of human proteins contributing to the two ES scores, there are also proteins that contribute only to the degree ES score or only to centrality ES score.

## 2 GSEA Analysis for Pathogen Groups

Supplementary Table S3 presents GSEA results for all pathogens with at least seven human protein interactors that are enriched for both GSEA analyses. We choose seven as a cutoff, since the remaining pathogens interact with three or fewer human proteins.

## References

1. Yu H, Kim PM, Sprecher E, Trifonov V, Gerstein M (2007) The importance of bottlenecks in protein networks: correlation with gene essentiality and expression dynamics. PLoS Computational Biology 3:e59.
