# Supplementary material for: The Landscape of Human Proteins Interacting with Viruses and Other Pathogens
Source: PLoS Pathog. 2008 Feb 15;4(2):e32. doi: 10.1371/journal.ppat.0040032 (PMC2242834; doi:10.1371/journal.ppat.0040032)
Supplement: Supplementary file 2 [file ppat.0040032.st001.pdf]

Table S1. Relative occurrences of four types of nodes in each of the three networks: Whole human PPI network (W), the human PPI network yielded by High-Throughput experiments (HT), and the human PPI network consisting only of Manually Curated PPIs (MC). The "Fraction" column defines the cutoff at which a protein is considered a hub or a bottleneck. The other columns represent the fraction of hub-bottleneck, non-hub-bottleneck, hub-non-bottleneck, and non-hub-non-bottleneck proteins in the network using that cutoff.

Network	Fraction	Hub-Bottleneck	Non-Hub-Bottleneck	Hub-Non-Bottleneck	Non-Hub-Non-Bottleneck
W	0.10	0.001	0.039	0.000	0.960
	0.20	0.003	0.078	0.001	0.918
	0.30	0.007	0.115	0.002	0.876
	0.40	0.012	0.155	0.010	0.824
HT	0.10	0.002	0.033	0.000	0.965
	0.20	0.004	0.066	0.000	0.930
	0.30	0.006	0.100	0.000	0.894
	0.40	0.009	0.132	0.000	0.859
MC	0.10	0.001	0.043	0.001	0.955
	0.20	0.003	0.086	0.002	0.909
	0.30	0.008	0.127	0.007	0.858
	0.40	0.012	0.173	0.021	0.794
