# Supplementary material for: The Landscape of Human Proteins Interacting with Viruses and Other Pathogens
Source: PLoS Pathog. 2008 Feb 15;4(2):e32. doi: 10.1371/journal.ppat.0040032 (PMC2242834; doi:10.1371/journal.ppat.0040032)
Supplement: Supplementary file 3 [file ppat.0040032.st002.pdf]

Table S2. Summary of GSEA results with and without human-HIV inter-actions for three networks: Whole human PPI network (W), the human PPI network yielded by High-Throughput experiments (HT), and the human PPI network consisting only of Manually Curated PPIs (MC). We report *p*-values only for the sets of human proteins in Figure 3. The "#proteins in group" column displays the total number of human proteins in that group. The "ES " column displays the enrichment score calculated by GSEA. The column titled "#proteins contributing" displays the number of proteins contributing to the ES score (see Methods for details.) The column titled "Jaccard's" lists the Jaccard coefficient between the two sets of proteins contributing to the ES score for degree and for centrality.

			Degree			Centrality			
Network	Protein Set	#proteins in group	ES	#proteins contributing	p-value	ES	#proteins contributing	p-value	Jaccards'
All Human-Pathogen PPIs									
W	Virus	1,029	0.79	563	$< 1 \times 10^{-6}$	0.83	349	$< 1 \times 10^{-6}$	0.51
	Multivirus	182	0.84	129	$< 1 \times 10^{-6}$	0.86	80	1.2×10^{-4}	0.60
	Bacteria	108	0.76	65	3×10^{-5}	0.89	31	2.3×10^{-4}	0.45
HT	Virus	466	0.68	156	$< 1 \times 10^{-6}$	0.83	108	1.5×10^{-3}	0.53
	Multivirus	98	0.65	36	0.03	0.86	31	0.1	0.68
	Bacteria	43	0.79	13	2×10^{-3}	0.89	12	0.02	0.66
MC	Virus	958	0.78	523	$< 1 \times 10^{-6}$	0.83	308	$< 1 \times 10^{-6}$	0.47
	Multivirus	174	0.83	119	$< 1 \times 10^{-6}$	0.86	85	1.9×10^{-5}	0.63
	Bacteria	100	0.73	66	3.4×10^{-4}	0.89	37	9.2×10^{-4}	0.51
Without Human-HIV PPIs									
W	Virus	499	0.80	263	$< 1 \times 10^{-6}$	0.85	195	$< 1 \times 10^{-6}$	0.59
	Multivirus	81	0.83	60	$< 1 \times 10^{-6}$	0.86	43	3.6×10^{-3}	0.66
HT	Virus	267	0.70	93	1×10^{-6}	0.84	66	6.1×10^{-6}	0.57
	Multivirus	46	0.72	21	0.02	0.86	18	0.07	0.70
MC	Virus	958	0.78	523	$< 1 \times 10^{-6}$	0.80	308	$< 1 \times 10^{-6}$	0.47
	Multivirus	174	0.83	119	$< 1 \times 10^{-6}$	0.85	85	1.3×10^{-3}	0.63
