# Supplementary material for: The Landscape of Human Proteins Interacting with Viruses and Other Pathogens
Source: PLoS Pathog. 2008 Feb 15;4(2):e32. doi: 10.1371/journal.ppat.0040032 (PMC2242834; doi:10.1371/journal.ppat.0040032)
Supplement: Supplementary file 4 [file ppat.0040032.st003.pdf]

Table S3. Summary of GSEA results for individual pathogen gorups for three networks: Whole human PPI network (W), the human PPI network yielded by High-Throughput experiments (HT), and the human PPI network consisting only of Manually Curated PPIs (MC). We report *p*-values only for the sets of human proteins in Figure 3. The "#proteins in group" column displays the total number of human proteins in that group. The "ES " column displays the enrichment score calculated by GSEA. The column titled "#proteins contributing" displays the number of proteins contributing to the ES score (see Methods for details.) The column titled "Jaccard's" lists the Jaccard coefficient between the two sets of proteins contributing to the ES score for degree and for centrality. We only report groups that are enriched for both degree and centrality.

All Human-Pathogen PPIs									
			Degree			Centrality			
Network	Protein Set	#proteins in group	ES	#proteins contributing	p-value	ES	#proteins contributing	p-value	Jaccards'
W	Adenovirus	59	0.79	34	2×10^{-4}	0.85	23	0.03	0.63
	Chlamydia	19	0.79	18	0.03	0.91	5	0.04	0.27
	EBV	121	0.72	61	3.7×10^{-4}	0.85	41	4.8×10^{-3}	0.57
	Hepatitis	93	0.81	47	$< 1 \times 10^{-6}$	0.91	33	5.8×10^{-5}	0.67
	Herpesvirus	54	0.80	32	1.1×10^{-4}	0.88	24	0.01	0.70
	HIV	671	0.79	358	$< 1 \times 10^{-6}$	0.81	218	4×10^{-6}	0.48
	Papillomavirus	94	0.79	66	1×10^{-6}	0.87	46	2.3×10^{-3}	0.67
	Polyomavirus	8	0.97	6	7×10^{-5}	0.96	5	0.03	0.83
	Pseudomonas	9	0.91	7	4.2×10^{-3}	0.97	8	0.02	0.88
	Sarcoma virus	35	0.81	31	1.1×10^{-3}	0.88	20	0.04	0.65
HT	EBV	64	0.76	17	8.9×10^{-4}	0.88	64	0.02	0.83
	Herpesvirus	22	0.84	6	4.6×10^{-3}	0.95	22	7.8×10^{-3}	0.83
MC	Adenovirus	57	0.79	29	6.4×10^{-5}	0.86	23	7.6×10^{-3}	0.73
	EBV	110	0.7	56	3.1×10^{-3}	0.85	38	5.9×10^{-4}	0.57
	Hepatitis	89	0.79	44	$< 1 \times 10^{-6}$	0.91	30	8×10^{-6}	0.64
	Herpesvirus	50	0.77	31	9.8×10^{-4}	0.84	24	0.02	0.72
	HIV	620	0.77	358	$< 1 \times 10^{-6}$	0.79	188	$< 1 \times 10^{-6}$	0.44
	Influenza	76	0.94	67	$< 1 \times 10^{-6}$	0.74	42	0.03	0.47
	Papillomavirus	90	0.76	70	1.5×10^{-5}	0.85	30	2.2×10^{-3}	0.41
	Polyomavirus	7	0.97	6	6×10^{-6}	0.97	5	0.02	0.83
	Pseudomonas	9	0.9	7	5.4×10^{-3}	0.94	7	0.03	1
	Sarcoma virus	34	0.79	21	2.1×10^{-3}	0.87	19	0.02	0.82
